# Increasing Importance of Genotype–Phenotype Correlations Associated with Common and Rare MEFV Gene Mutations in FMF Patients in the Last Thirty Years

**DOI:** 10.3390/jcm14030712

**Published:** 2025-01-22

**Authors:** Sema Yildirim, Hayrunnisa Bekis Bozkurt, Muferet Erguven

**Affiliations:** 1 Department of Pediatrics, Göztepe Prof. Dr. Süleyman Yalçın City Hospital, 34722 İstanbul, Turkey; 2 Department of Pediatrics, Faculty of Medicine, Kafkas University, 36000 Kars, Turkey; hayrunisabekis@hotmail.com; 3 Division of Rheumatology, Department of Pediatrics, Faculty of Medicine, Düzce University, 81620 Düzce, Turkey; muferete@yahoo.com

**Keywords:** disease severity, Familial Mediterranean Fever, genotype, phenotype, rare mutations

## Abstract

**Background/Objectives**: Studies have shown that some mutations, especially M694V, are correlated with renal RI and/or AA. There are limited data about rare mutations on severity of the disease and RI. Today, evaluating genotype–phenotype correlations in rare mutations is important to better understand FMF. We aimed to evaluate clinical, demographic and genetic changes and genotype–phenotype correlations in pediatric patients with FMF over thirty years as well as the importance of the rare mutations. **Methods**: A total of 2765 pediatric patients with FMF were included in this study. Genetic results were firstly divided into ten groups including rare mutations. Rare mutations were seen in 2% of all patients and divided into eight groups. **Results**: There was a significant increase in compound heterozygous mutations, E148Q het/hom, R202Q het/hom, complex mutations and rare mutations in the last decade. RI wo AA was 5.8% and AA was 1% in the patients with rare mutations. While M694V and compound het with M694V were positively correlated with severe PRAS, E148Q and V726A were negatively correlated with severe PRAS (*p* < 0.05, R = 0.137, R = −0.077, R= −0.05, respectively). Although K695R mutation was negatively correlated with severe PRAS (*p* < 0.05, R = −0.04), the rate of RI was 20%. Although the rare mutation R761H was negatively correlated with severe PRAS (*p* < 0.05, R = −0.051), the colchicine resistance rate was 8.3%. **Conclusions**: It may be misleading for clinicians that mutations which have increased in frequency over the years are clinically mild. RI and AA rates in rare mutations are not less than the related rates in common mutations.

## 1. Introduction

Familial Mediterranean Fever (FMF) is an autosomal recessive autoinflammatory disease characterized by recurrent self-limiting episodes. These episodes typically involve attacks of polyserositis, abdominal pain and fever [1]. Symptoms of FMF usually begin in childhood, and 90% of the symptoms start before the age of 20. People are diagnosed with FMF based on clinical signs and symptoms [2]. Also, the interest in mutations of the Mediterranean Fever (MEFV) gene, located on the short arm of chromosome 16, has increased in recent years, with more than 150 mutations reported [1,2]. Most common genetic mutations are identified in the alleles of M694V, M680I, V726A, E148Q and M694I. Most known rare mutations are A744S, R761H, P396S, K695R and F479L alleles [3]. It is widely recognized that the disease tends to progress more severely in cases with mutations like M694V and M680I. In contrast, those with mutations such as V726A and E148Q typically experience a milder form of the disease. The most serious complication of the disease is end-stage renal failure, which occurs due to accumulation of amyloidosis (AA) [4]. In some cases, renal involvement (RI) may occur without the development of AA. It is recognized that inflammation during attacks, along with the persistence of subclinical inflammation between attacks, serves as the primary pathological mechanism. Approximately one in ten patients with FMF who are receiving colchicine treatment will develop RI [5]. Studies have shown that some mutations, especially M694V mutations, are correlated with RI and/or AA. There are limited data about rare mutations on the severity of the disease and RI. Today, evaluating genotype–phenotype correlations in rare mutations is important to better understand FMF.

The aim of this study is to evaluate clinical, demographic and genetic changes and genotype–phenotype correlations in pediatric patients with FMF over the past thirty years and to assess the importance of rare mutations.

## 2. Material and Methods

### 2.1. Participants

Patients diagnosed with FMF based on the Tel-Hashomer criteria [6] or Yalçınkaya criteria (7) in two university hospitals between June 1990 and December 2019 were included in this study. This study included two hospitals, one located in İstanbul, the most populous city in western Turkey, classified as Region 1. The other hospital is located in Kars, in eastern Turkey, where FMF is more frequent, and it is classified as Region 2. Both hospitals are important tertiary hospitals in their respective regions. The clinical findings (at/after the time of diagnosis) and demographic characteristics of the patients, along with their laboratory results from the most recent episode and their conditions during the follow-up period were obtained from the archived patient files. Patients who did not attend follow-up appointments were excluded from this study based on specific medical criteria. Additionally, patients with congenital heart disease, congenital renal disease, and chronic lung or liver disease, as well as those who did not have a definitive diagnosis and presenting with syndromic findings or those diagnosed with immunodeficiency were not included in this study. The age at diagnosis, the age at symptom onset, the duration of diagnostic delay, gender, clinical complaints, symptoms and signs, family history, the presence of the genetic test results and the severity of the disease at the last visit were recorded from patients’ files. Severity of the disease was assessed using the PRAS score. This scoring system assesses severity of the disease based on the following criteria: “Age of onset (<6 years is 4 points; 6–10 years is 3 points; and 11–20 years is 2 points), number of attacks per month (<1 is 1 point; 1–2 is 2 point; and >2 is 3 points), presence of arthritis (acute is 2 points and protracted is 3 points), presence of erysipel like erythema (2 points), presence of amyloidosis (3 points) and colchicine dose (1 mg/day is 1 point; 1.5 mg/day is 2 points; 2 mg/day is 3 points, and >2 mg/day is 4 points)” [7,8]. All results were evaluated according to three decades and regions.

### 2.2. Treatment of the Patients

Colchicine treatment was initiated promptly upon diagnosis for all patients who met the Tel-Hashomer or Yalçınkaya diagnostic criteria. The patients were closely monitored every three or four months, and the colchicine dosage was adjusted as needed, with a maximum of 2 mg per day. During follow-up assessments, we evaluated drug compliance, the occurrence and severity of any attacks compared to previous episodes, and identified potential triggers. Interleukin-1 (IL-1) antagonist was used in colchicine-resistant-patients with FMF. Colchicine resistance was defined as experiencing more than six attacks per year or more than three attacks within 4 to 6 months, despite at least one year of colchicine treatment [9]. The diagnosis of amyloidosis was established through histological analysis of biopsy samples. If the results indicated the presence of congophilic fibrillar amyloid deposits, the diagnosis was classified as AA. Tissue samples for this analysis were obtained from the kidneys, rectum and spleen. RI wo AA referred to patients with nephritic or nephrotic proteinuria and/or hematuria [4,10].

### 2.3. Genetic Analysis

MEFV mutations were analyzed with polymerase chain reaction (PCR) in conjunction with restriction fragment length polymorphism (PCR–RFLP) or the reverse hybridization assay. The genetic results were categorized into ten groups; M694V het/hom, compound mutations (with and without M694V), R202Q het/hom, E148Q het/hom, M680I het/hom, V726A het/hom, complex heterozygous (M694V/E148Q/R202Q, V726A/M680I/P396S, A744S/R761/E148Q, …, etc.), no mutations and rare mutations. Rare mutations were divided into eight groups: A744S het/hom, R761H het/hom, P396S het/hom, K695R het/hom, M694I het/hom, F479L het/hom, G304R het/hom and other mutations.

### 2.4. Statistical Analysis

The Shapiro–Wilk and Kolmogorov–Smirnov normality tests were used to determine the distribution patterns of the variables. Continuous data are presented as mean (S.D.) and median, accompanied by the minimum and maximum values. The Mann–Whitney *U* test and Student’s *t*-test were used for comparisons between more than two groups and two groups of continuous variables, respectively. The chi-squared test was used to analyze categorical variables. Spearman’s correlation test was utilized to assess the correlation between various data reflecting clinical severity (PRAS score, attack frequency, RI wo AA, AA and colchicine resistance), clinical findings and genetic outcomes. Linear regression analysis was performed to evaluate the correlation between PRAS score and genetic results increasing over the years. The Kaplan–Meier analysis was conducted to assess the effects of gender, region, variants of MEFV mutations, PRAS score disease and colchicine resistance on the progression of amyloidosis over the years. A *p* value of < 0.05 was considered as statistically significant. The statistical analysis was conducted using the Statistical Package for the Social Science (SPSS) software version 26.0 (SPSS, Chicago, IL, USA).

Approval was obtained from the local university Ethics Committee (2019/80576354-050-99/81) prior to the initiation of the experiment. This study was conducted in accordance with the principles outlined in the Helsinki Declaration.

## 3. Results

**Figure 1:**
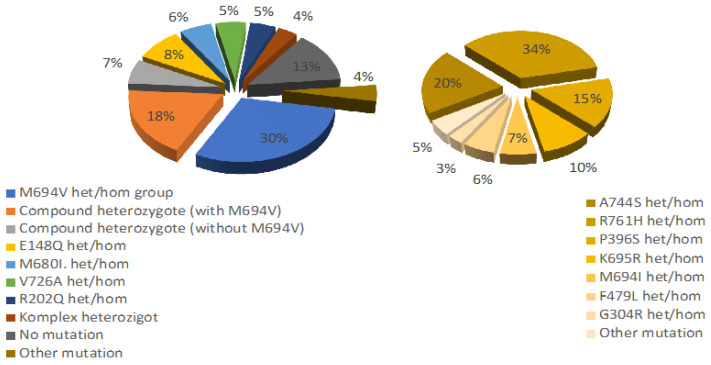
Distribution of the genetic mutations.

**Figure 2:**
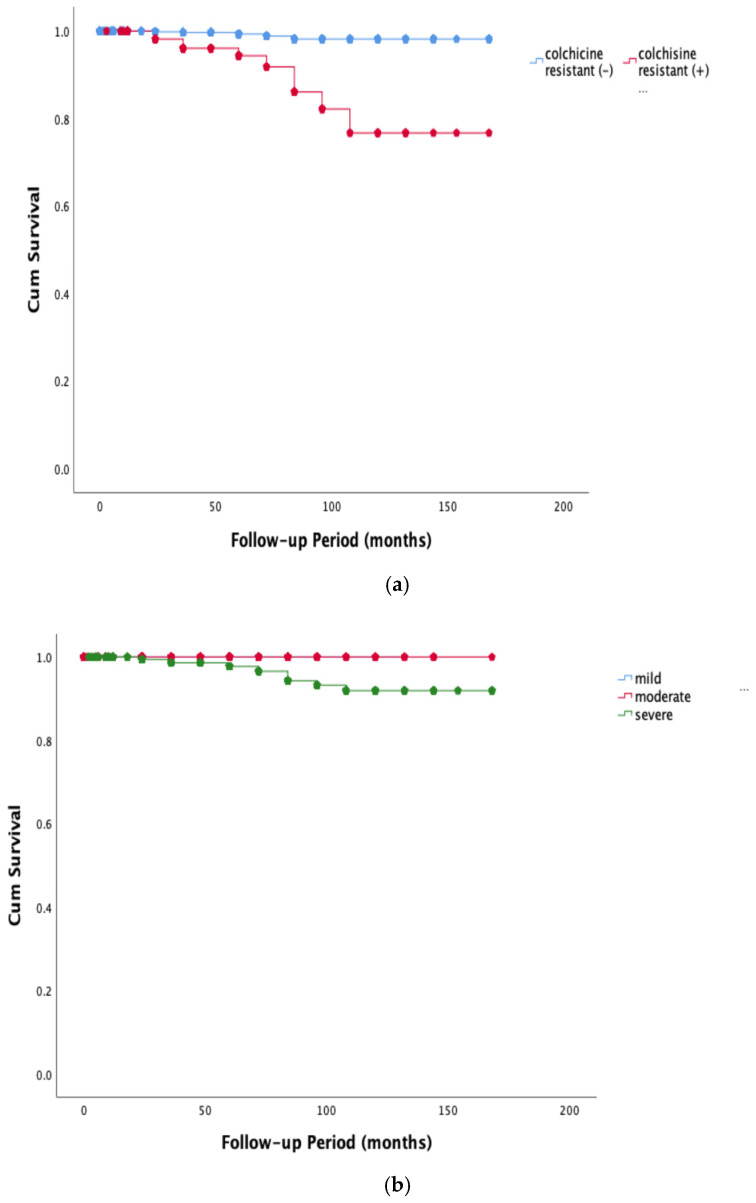
(**a**) A comparison of amyloidosis development according to the presence of colchicine resistance. (**b**) A comparison of amyloidosis development according to the PRAS score severity of disease.

This study comprised 2765 patients from two university hospital centers. A total of 372 patients were excluded due to lack of data. A total of 49.9% (n = 1379) of the patients were male. Age of the participants during the study time was 14.23 ± 3.6 years. The consanguinity rate was observed to be higher in Region 1 compared to Region 2 (29.3%, 19.3%, respectively, *p* = 0.001). A comparison of clinical, demographic and genetic results of the patients is presented in Table 1. The delay in diagnosis decreased significantly over the years (*p* = 0.04). In the last decade, the number of family members with FMF and the rate of genetic diagnosis increased significantly (*p* = 0.001, *p* < 0.001, respectively). The most commonly observed clinical findings included abdominal pain (80.5%), fever (60%) and arthralgia (49.3%). Although the order of frequency remained consistent over the years, both chest pain and arthralgia showed a significant increase in the last decade (*p* = 0.002, *p* = 0.001, respectively). AA was 1%, while the rate of RI wo AA was 5.7%. A significant decrease in RI wo AA has occurred in the last decade (*p* = 0.001). Severe PRAS was highest in the second decade, but in the third decade, still one-third of the patients had severe PRAS. The colchicine resistance rate was 5.7% (Table 2). M694V het/hom and compound het with M694V accounted for about half of the cases. In rare mutations, the most common was A744S het/hom (n = 36) and the second most frequent was R761H (n = 21), P396S (n = 16) and K695R (n = 10) (Figure 1). There was a significant increase in compound heterozygous mutations, E148Q, R202Q, complex mutations and rare mutations in the last decade (*p* = 0.06, *p* < 0.001, *p* < 0.001, *p* < 0.001, *p* = 0.005, respectively) (Table 3). The rate of RI wo AA was 5.8% and the rate of AA was 1% in the patients with rare mutations. Delays in diagnosis were lower in the last decade and there was no significance in other clinical findings and laboratory results between decades (*p* > 0.005) (Table 4). There was a positive correlation between the presence of M694V het/hom and the occurrence of monoarthritis/polyarthritis. Furthermore, A744S demonstrated a positive association with symptoms of nausea/vomiting, while P396S/K695R did not present any associated symptoms (*p* < 0.005, R = 0.081, R = 0.042, R = 0.042, R = 0.038, R = 0.044, respectively). Conversely, a negative correlation was observed between E148Q and chest pain, monoarthritis; V726A and constipation; and complex heterozygotes and polyarthritis (*p* < 0.005, R = −0.041, R = −0.042, R = −0.043, R = −0.069) (Table 5). When examining the relationship between genetic mutations and disease severity, only M694V het/hom mutation was positively correlated with RI wo AA, AA and colchicine resistance (*p* < 0.005, R = 0.074, R = 0.063, R = 0.06). In contrast, E148Q het/hom mutation and V726A het/hom mutation were severely negatively correlated with the severity of PRAS (*p* < 0.005, R = −0.077, R = −0.05). Although the K695R mutation was negatively correlated with severe PRAS (*p* < 0.005, R = −0.04), the ratio of renal involvement was 20%. Although the rare mutation R761H was severely negatively correlated with PRAS (*p* < 0.005, R = −0.051), a colchicine resistance of 8.3% was present. A744S mutation was detected only in the AA case among the rare mutations (Table 6). Rare mutations whose frequency increased over the years had a weak negative correlation with PRAS score and no effect on complex mutations, and this correlation was minimal in R761H and compound mutations (R2 = 0.005, R2 = 0.001). In the Kaplan–Meier analyses conducted to evaluate the factors effecting development of amyloidosis over the years, statistically significant differences were observed when stratified by colchicine resistance and PRAS score severity of disease (Log Rank test, *p* < 0.001, *p* = 0.022, respectively), as shown in Figure 2a,b. Regions, gender and variations in MFV mutations, however, did not show statistically significant differences. (Log Rank test, *p* = 0.437, *p* = 0.638, *p* = 0.341, respectively).

## 4. Discussion

FMF is diagnosed based on clinical findings, family history and laboratory tests. Disease-related mutations in the gene consisting of 10 exons began to be identified since 1997. Molecular genetic tests have been gaining increasing importance in FMF, which show common clinical features with other auto-inflammatory diseases defined in recent years. To investigate the genotype–phenotype relationship by revealing common MEFV gene mutations and rare mutations of the disease severity, risk of amyloidosis and colchicine resistance are important in the prediction of the disease course.

In the present study, a significant increase was detected in compound heterozygous mutations, E148Q het/hom, R202Q het/hom, complex mutations and rare mutations over the years. Although there was a decrease in RI wo AA, AA rates and delay in diagnosis, colchicine resistance increased compared to the values detected in the first decade. Although types of rare mutations with increasing frequency were negatively correlated with severe PRAS and positively correlated with fainter/no symptoms, RI wo AA was 5.8% and AA was 1% in rare mutations.

Five major mutations, M694V, V726A, M694I, M680I and E148Q accounted for 74% of FMF genetic mutations identified in Armenians, Turks, Arabs and Jews [11]. As stated previously, the M694V het/hom mutation (30%) was the most detected mutation among different ethnic groups in the present study [12,13]. Compound heterozygous mutations (24.7%) were the second most common detected mutation in the present study. Compound heterozygous mutations were also seen at significant rates over the years between 18.6 and 30.1% [14,15]. The third most common mutation identified in this study was E148Q (8.4%), which has also been reported as the most common mutation in several previous studies from Turkey [14,16]. M680I, V726A and R202Q were other common mutations detected. The R202Q variation has been reported as a polymorphism in previous studies. However, some researchers emphasize that the R202Q mutation is linked to the inflammatory phenotype and could be a disease-causing mutation [1,17,18,19]. Most of the rare mutations were R761H, A744S, and P396S. Numerous studies have reported that these rare mutations range between 0.5 and 4% [15,20,21,22]. The mutation rates observed in this study are closely consistent with findings from both international and national research [1,23,24,25].

Abdominal pain, fever and arthralgia are the most common clinical symptoms associated with FMF. Additional symptoms may include chest pain, nausea, vomiting and fibromyalgia. The clinical findings presented in this study are compatible with the existing literature [1,21]. The order of frequency did not change over the years, but chest pain and arthralgia increased. In recent years, FMF patients with chest pain have started to attract attention. In the present study, compound heterozygous mutation with M694V was positively correlated with chest pain, and E148Q mutation had a negative correlation with chest pain. In addition, the highest rate of chest pain was detected in the M694I mutation, which is one of the rare mutations (28.6%: 2 out of 7 patients). The studies indicated that chest pain was frequently seen in the R202Q mutation or compound mutations accompanied by the R202Q mutation, but no correlation was found [21]. In another study, this rate was 18.6% for E148Q, but data on rare mutations were insufficient [21,26]. In the present study, chest pain was 13.6% in E148Q mutation, 18.6% in R202Q mutation and 23.4% in compound heterozygous mutation with M694V. Although the frequency of arthralgia/arthritis increased over the years, there was a decrease in disease severity and rate of RI wo AA. This can be explained by the decrease in the delay in diagnosis and the increase in our experience with the disease. Similar to the present study, numerous studies reported a correlation between the M694V mutation and arthritis/arthralgia [27,28]. Abdominal pain, arthralgia and fever were observed most frequently in rare mutations, but we could not detect a correlation with these common findings.

Numerous studies have shown that while the M694V mutation is associated with severe disease, the E148Q, R202Q and V726A mutations are associated with milder forms of the disease [1,29,30]. Additionally, various studies have indicated that compound heterozygous mutations are typically linked to milder disease compared to homozygous mutations [21,31]. However, in this study, compound heterozygous M694V mutations involving M694V are significantly correlated with severe PRAS. A negative correlation was observed between E148Q het/hom and the V726A het/hom, while a positive correlation was found between the M694V het/hom and severe PRAS. Although the rate of severe PRAS was 43% in R202Q het/hom mutations, no significance could be obtained.

In a study conducted in Israel, 10 patients with K695R and 17 patients with A744S mutations had clinically mild symptoms [32]. Söylemezoğlu et al., found that A744S, R761H and P396S were the most common rare mutations, similar to the findings of this study. They reported no differences in disease severity between patients with rare mutations and those with common mutations in a single allele form. Additionally, the number of patients with single allele mutations of R761H, K695R and F479L was insufficient for mutation-specific statistical analysis. The correlation between these mutations and clinical symptoms was not detailed [22]. In this study, a positive correlation was found between P396S and/or K695R and the absence of symptoms or faint signs. These patients had applied with an insidious clinic over the years. Moreover, K695R was negatively correlated with severe PRAS, but the RI wo AA rate was 6.3% and 20% for P396S and K695R, respectively.

AA is the most important complication of FMF. Prevention of AA is the most important indicator of successful follow-up and management of FMF. An international multicenter study conducted in 2007, which included data from Turkey, found that the incidence of amyloidosis was 10.4%. However, a subsequent multicenter study carried out in Turkey in 2014 reported the incidence at 8.6%, while a study conducted in 2021 showed a much lower incidence of 0.2% [27,33,34]. AA rates decreased from 1.7% to 0.9% in the last two decades and 1% in total. The rate of RI wo AA was 5.7% and decreased significantly over the years in the present study. This was significantly positive for follow-up and treatment. These rates were similar in rare mutations as well as common mutations (for RI wo AA and AA, 5.8%, 1%; 5.7%, 1%, respectively). Data on the incidence of AA in rare mutations are insufficient, and most studies have reported that it has been associated with mild disease [32]. Consistent with the literature, the strongest positive correlation with AA was found in the M694V mutation [28]. A744S was the only rare mutation in patients with AA. Although the rate of A744S was around 3.5–6.5% in studies conducted in Africa, AA was not reported [35]. A744S also showed a positive correlation with nausea and vomiting.

Colchicine resistance is another important issue. Colchicine is a proven treatment to prevent AA in the management of FMF. Today, 5% to 10% of colchicine resistance is reported despite the most successful follow-up [36]. National studies report that these rates vary between 2.7 and 6.6% and are compatible with the results of the present study [16,37]. This study revealed that although colchicine resistance showed a partial decrease in the last decade, it increased significantly compared to the first decade and was 5.7% in total. In rare mutations, colchicine resistance was found in 3.8%. Although the R761H mutation, which is one of the rare mutations, was negatively correlated with severe PRAS, colchicine resistance was 8.3%. In the study by Çakan et al., only 1 of 19 colchicine resistant patients had the R761H/M694V compound mutation [38]. Colchicine resistance and AA have been reported in compound mutations (M694V/R761H) rather than single mutations [38]. In another recent study, rare mutations were not found in 65 patients with colchicine resistance who received biologics [39].

In conclusion, clinically mild clinical presentations of mutations, which have increased in frequency over the years but are seen more rarely, may be misleading for clinicians. Although the rate of delay in diagnosis decreases, colchicine resistance is an important problem with a rate of 5.7%. It should be kept in mind that there was a positive correlation with nausea and vomiting in patients with A744S het/hom mutations and with arthritis and severe PRAS in M694V patients. The rates of RI and AA in rare mutations were not less than the related rates in common mutations. Patients with rare mutations should be followed closely by clinicians.

## Figures and Tables

**Table 1:** A comparison of demographic, clinical and genetic results of patients according to their regions.

	Region 1 (İstanbul) n = 2362	Region 2 (Kars) n = 403	*p*
Age at the time of diagnosis (years) ^a^	9.03 ± 4.31	8.93 ± 4.2	0.666
Age at the study time (years) ^a^	15.84 ± 5.91	17.49 ± 6.06	0.377
Gender (male) ^b^	1167 (49.4)	212 (52.6)	0.235
Delay in diagnosis (months) ^a^	29.94 ± 31.7	32.04 ± 31.89	0.298
Follow up period (months) ^a^	45.57 ± 33.97	36.33 ± 38.96	0.227
Consanguinity ^b^	455 (19.3)	118 (29.3)	**0.001**
FMF in the family ^b^	1127 (47.7)	224 (55.6)	0.104
AA ^b^	24 (1)	5 (1.2)	0.676
Resistance to colchicine ^b^	140 (5.9)	17 (4.2)	0.171
M694V het/hom group ^b^	629 (30.5)	92 (27.2)	0.962
Compound heterozygote (with M694V) ^b^	370 (17.9)	65 (19.2)	0.577
Compound heterozygote (without M694V) ^b^	146 (7.1)	11 (3.3)	0.108
E148Q het/hom ^b^	170 (8.2)	32 (9.5)	0.455
M680I. het/hom ^b^	118 (5.7)	21 (15.1)	0.606
V726A het/hom ^b^	103 (79.8)	26 (20.2)	0.348
R202Q het/hom ^b^	88 (79.3)	23 (20.7)	0.274
Complex heterozigot ^b^	70 (3.4)	16 (4.7)	0.061
Rare mutations ^b^	86 (4.2)	18 (5.35)	0.421
A744S het/hom ^b^	18 (0.8)	3 (0.7)	0.422
R761H het/hom ^b^	26 (1.1)	10 (2.5)	0.707
P396S het/hom ^b^	14 (0.6)	2 (0.5)	0.109
K695R het/hom ^b^	10 (0.4)	0	0.191

^a^: Mean ± SD, ^b^: n (%), FMF: Familial Mediterranean Fever, AA: Amyloidosis.

**Table 2:** Characteristics, clinical signs and resistance to colchicine.

	1990–1999	2000–2009	2010–2019	Total	*p*_1_	*p*_2_
Age at the time of diagnosis (years) ^a^	9.15 ± 3.61	9.1 ± 4.01	8.89 ± 4.3	8.95 ± 4.22	0.635 *	0.530 **
Age at the study time (years) ^a^	17.12 ± 4.74	14.75 ± 3.7	11.2 ± 4.23	14.23 ± 3.6	0.001 *	0.001 **
Gender (male) ^b^	31 (50)	326 (49.1)	1029 (50.5)	1379 (49.9)	0.829 *	0.89 **
Delay in diagnosis (months) ^a^	35.1 ± 31.26	34.78 ± 32.83	30.6 ± 31.5	31.7 ± 31.8	0.04 *	0.045 **
Follow up period (months) ^a^	87.28 ± 53.11	61.79 ± 37.97	28.2 ± 20.87	44.03 ± 35.01	0.036 *	0.002 **
Consanguinity ^b^	6 (9.7)	147 (22.1)	420 (20.6)	573 (20.7)	0.066 ^#^	0.686 ^#^
FMF in the family ^b^	17 (27.4)	314 (47.3)	1020 (50)	1351 (48.9)	**0.001 ^#^**	0.632 ^#^
Presence of the genetic result ^b^	25 (40.3)	592 (89.2)	1785 (87.5)	2402 (86.9)	**<0.001 ^#^**	0.545 ^#^
**Clinical signs during the first attack at/after the time of diagnosis ^b^**					*p*_1_ ^#^	*p*_2_ ^#^
Abdominal pain	33 (53.2)	568 (85.5)	1625 (79.7)	226 (80.5)	**0.001**	0.790
Fever	25 (40.3)	401 (60.4)	1234 (60.5)	1660 (60)	**0.006**	0.086
Nausea/vomiting	7 (11.3)	124 (18.7)	399 (19.6)	530 (19.2)	0.247	0.206
Diarrhea	2 (3.2)	76 (11.4)	240 (11.8)	318 (11.5)	0.115	0.204
Chest pain	4 (6.5)	154 (23.2)	386 (18.9)	544 (19.7)	**0.002**	0.504
Arthralgia	17 (27.4)	308 (46.4)	1037 (50.9)	1362 (49.3)	**0.001**	0.545
Monoarthritis	4 (6.5)	88 (13.3)	248 (12.2)	340 (12.3)	0.278	0.233
Polyarthritis	4 (6.5)	47 (7.1)	120 (5.9)	171 (6.2)	0.539	0.321
Fibromyalgia	2 (3.2)	56 (8.4)	177 (8.7)	235 (8.5)	0.316	0.230
Erysipel	1 (1.6)	19 (2.9)	50 (2.5)	70 (2.5)	0.757	0.799
Scrotal pain	0	5 (0.8)	31 (1.5)	36 (1.3)	0.209	0.124
Constipation	0	6 (0.9)	24 (1.2)	30 (1.1)	0.593	0.422
RI wo AA	6 (9.7)	63 (9.5)	88 (4.3)	157 (5.7)	**0.001**	**0.001**
AA	0	11 (1.7)	18 (0.9)	29 (1)	0.168	0.143
**PRAS Score ^b^**						
Mild	29 (46.8)	101 (15.2)	545 (26.7)	675 (24.4)		
Modarate	23 (37.1)	201 (30.3)	808 (39.6)	1032 (37.3)	**0.001**	**0.001**
Severe	10 (16.1)	362 (54.5)	686 (33.6)	1058 (38.3)		
**Laboratory results at the time of last attack**					*p*_1_	*p*_2_
WBC (×10^3^/mm^3^) ^a^	8.4 ± 1.14	9.8 ± 6.4	9.18 ± 4.87	9.33 ± 5.35		
Hb (g/dL) ^a^	13.2 ± 1.09	12.05 ± 1.22	12.05 ± 1.22	12.18 ± 1.3		
Plt (×10^3^/mm^3^) ^a^	293.15 ± 66.4	308.28 ± 97.55	308.97 ± 89	308.49 ± 90.8	0.325 *	0.371 **
CRP (mg/dL) ^a^	12.42 ± 12.26	14.78 ± 12.32	10.42 ± 12.13	11.66 ± 12.26		
ESR (mm/h) ^a^	33.74 ± 28.03	31.44 ± 26.22	30.87 ± 22.44	28.97 ± 23.3		
**Resistance to colchicine ^b^**	2 (3.2)	56 (8.4)	99 (4.9)	157 (5.7)	**0.002 ^#^**	0.053 ^#^

*p*_1_: between three groups, *p*_2_: between last two decades, ^a^: Mean ± SD, ^b^: n (%), FMF: Familial Mediterranean Fever, RI wo AA: renal involvement without amyloidosis, AA: amyloidosis, WBC: white blood cell; Hb: hemoglobin, Plt: platelets, CRP: C-reactive protein; ESR: erythrocyte sedimentation rate; * Mann–Whitney U test, ** Student’s *t*-test, ^#^ chi-squared test.

**Table 3:** Distribution of genetic mutations according to decade.

	1990–1999	2000–2009	2010–2019	Total	*p*_1_ *	*p*_2_ *
M694V het/hom group	9 (36)	268 (45.3)	444 (24.9)	721 (30)	**<0.001**	**<0.001**
Compound heterozygote (with M694V)	4 (16)	90 (15.2)	341 (19.1)	435 (18.1)	**0.019**	0.054
Compound heterozygote (without M694V)	4 (16)	22 (3.7)	131 (7.3)	157 (6.5)	**0.01**	**0.006**
E148Q het/hom	1 (4)	17 (2.9)	184 (10.3)	202 (8.4)	**<0.001**	**<0.001**
M680I. het/hom	1 (4)	44 (7.4)	94 (5.3)	139 (5.8)	0.055	0.265
V726A het/hom	0	43 (7.3)	86 (4.8)	129 (5.4)	**0.012**	0.259
R202Q het/hom	1 (4)	4 (0.7)	106 (5.9)	111 (4.6)	**0.001**	**<0.001**
Complex heterozigot	1 (4)	6 (1)	79 (4.4)	86 (3.6)	**<0.001**	**<0.001**
Rare mutation	0	14 (2.4)	90 (5)	104 (4.3)	**0.014**	**0.005**
No mutation	4 (16)	79 (13.3)	235 (13.2)	318 (13.2)	0.089	0.067

*p*_1_: between three groups, *p*_2_: between last two decades, * chi-squared test.

**Table 4:** Evaluation of clinical findings, laboratory results and resistance to colchicine of patients with rare mutations during follow-up (n = 104).

	2000–2009	2010–2019	Total	*p*
Age at the time of diagnosis (years) ^a^	10.35 ± 3.98	8.84 ± 4.16	9.12 ± 4.15	0.652 ^#^
Gender (male) ^b^	6 (31.6)	43 (50.6)	55 (52.9)	0.133 ^#^
Delay in diagnosis (months) ^a^	28.94 ± 30.41	23.6 ± 20.3	24.89 ± 23.015	**0.014 ^#^**
Follow up period (months) ^a^	52.56 ± 47.9	22.28 ± 12.73	36.06 ± 36.7	**0.001 ^#^**
Consanguinity ^b^	1 (5.3)	18 (21.2)	19 (18.3)	0.105 ^##^
FMF in the family ^b^	9 (47.4)	35 (41.7)	44 (42.7)	0.650 ^##^
Presence of attack at the time of admission ^b^	9 (47.4)	39 (45.9)	48 (46.2)	0.906 ^##^
**Clinical signs ^b^**				*p* ^##^
Abdominal pain	15 (78.9)	68 (80)	83 (79.8)	0.918
Fever	8 (42.1)	40 (47.1)	48 (46.2)	0.695
Nausea/vomiting	3 (15.8)	18 (21.2)	21 (20.2)	0.597
Diarrhea	0	6 (7.1)	6 (5.8)	0.233
Chest pain	2 (10.5)	12 (14.1)	14 (13.5)	0.678
Arthralgia	7 (36.8)	44 (51.8)	51 (49)	0.239
Monoarthritis	4 (21.1)	10 (11.8)	14 (13.5)	0.284
Polyarthritis	0	0	0	NA
Fibromyalgia	2 (10.5)	7 (8.2)	9 (8.7)	0.748
Erysipel	0	2 (2.4)	2 (1.9)	0.366
Scrotal pain	0	1 (1.2)	1 (1)	0.635
Constipation	0	0	0	NA
RI wo AA ^b^	1 (5.3)	5 (5.9)	6 (5.8)	0.917
AA ^b^	0	1 * (1.2)	1 (1)	0.635
**PRAS Score ^b^**				
Mild	4 (21.1)	29 (34.1)	33 (31.7)	
Modarate	6 (31.6)	38 (44.7)	44 (42.3)	0.062
Severe	9 (47.4)	18 (21.2)	27 (26)	
**Laboratory results at the time of attack**				*p* ^#^
WBC (×10^3^/mm^3^) ^a^	9.63 ± 4.76	11 ± 3.08	10.6 ± 3.25	0.432
Hb (g/dL) ^a^	12.5 ± 0.7	12.33 ± 1.75	12.37 ± 1.5	0.291
Plt (×10^3^/mm^3^) ^a^	340.62 ± 125.84	310.9 ± 113.24	316.36 ± 115.47	0.527
CRP (mg/dL) ^a^	14.7 ± 9.87	15.5 ± 10.01	15.5 ± 10	0.985
ESR (mm/h) ^a^	27.92 ± 20.60	24.13 ± 18.25	24.83 ± 18.61	0.878
**Resistance to colchicine ^b^**	2 (10.5)	2 (2.4)	4 (3.8)	0.094 ^##^

^a^: Mean ± SD, ^b^: n (%), ^#^: Student-*t* test, ^##^: Chi-square test, ^*^: A744S mutation.

**Table 5:** Analysis of the Correlation between Genetic Results and Clinical Findings.

	Fever	Abdominal Pain	Chest Pain	Arthralgia	Monoarthritis	Poliarthritis	Nause-Vomiting	Diarrhea	Erisipel	Fibromyalgia	Constipation	Scrotal Pain	No Symptoms
M694V het/hom group	453 (62.8)	587 (81.4)	150 (20.8)	375 (52)	121 (16.8) *^,d^	57 (7.9) *^,g^	169 (23.4)	88 (12.2)	24 (3.3)	70 (9.7)	5 (0.7)	11 (1.5)	28 (3.9) **^,k^
Compound heterozygote (with M694V)	290 (66.7) *^,a^	345 (79.3)	102 (23.4) *^,b^	208 (47.8)	55 (12.6)	22 (5.1)	97 (22.3)	52 (12)	13 (3)	34 (7.8)	5 (1.1)	4 (0.9)	27 (6.2)
Compound heterozygote (without M694V)	85 (54.1)	127 (80.9)	40 (25.5)	78 (49.7)	11 (7)	9 (5.7)	50 (31.8)	23 (14.6)	4 (2.5)	10 (6.4)	0	1 (0.6)	9 (5.7)
E148Q het/hom	127 (62.9)	169 (83.7)	28 (13.9) **^,c^	107 (53)	15 (7.4) **^,e^	10 (5)	60 (29.7)	21 (10.4)	1 (0.5)	17 (8.4)	2 (1)	1 (0.5)	9 (4.5)
M680I. het/hom	86 (61.9)	109 (78.4)	30 (21.6)	67 (48.2)	17 (12.2)	10 (7.2)	40 (28.8)	11 (7.9)	4 (2.9)	6 (4.3)	3 (2.2)	1 (0.7)	9 (6.5)
V726A het/hom	74 (57.4)	100 (77.5)	19 (14.7)	70 (54.3)	8 (6.2) **^,f^	8 (6.2)	35 (27.1)	14 (10.9)	3 (2.3)	8 (6.2)	4 (3.1) **^,j^	4 (3.1)	10 (7.8)
R202Q het/hom	49 (57)	74 (86)	16 (18.6)	37 (43)	7 (8.1)	5 (5.8)	20 (23.3)	12 (14)	2 (2.3)	8 (6.2)	0	2 (2.3)	5 (5.8)
Complex heterozigot	70 (63.1)	88 (79.3)	22 (19.8)	49 (44.1)	7 (6.3)	1 (0.9) **^,h^	32 (28.8)	15 (13.5)	2 (1.8)	15 (13.5)	1 (0.9)	2 (1.8)	8 (7.2)
A744S het/hom	11 (52.4)	19 (90.5)	3 (14.3)	7 (33.3)	2 (9.5)	0	10 (47.6) *^,i^	1 (4.8)	0	2 (9.5)	0	0	1 (4.8)
R761H het/hom	17 (47.2)	28 (77.8)	3 (8.3)	19 (52.8)	6 (16.7)	0	6 (16.7)	3 (8.3)	0	2 (5.6)	0	1 (2.8)	3 (8.3)
P396S het/hom	6 (37.5)	11 (68.8)	3 (18.8)	7 (43.8)	3 (18.8)	0	6 (37.5)	1 (6.3)	1 (6.3)	2 (12.5)	0	0	3 (18.8) *^,l^
K695R het/hom	5 (50)	7 (70)	2 (20)	6 (60)	0	0	2 (20)	1 (10)	0	1 (10)	0	0	2 (20) *^,m^
M694I het/hom	2 (28.6)	6 (85.7)	2 (28.6)	5 (71.4)	1 (14.3)	0	1 (14.3)	0	0	2 (28.6)	0	0	1 (14.3)
F479L het/hom	2 (33.3)	5 (83.3)	0	5 (83.3)	0	0	1 (16.7)	0	0	0	0	0	1 (16.7)
G304R het/hom	2 (66.7)	3 (100)	0	1 (33.3)	1 (33.3)	0	2 (66.7)	0	0	0	0	0	0

*: positive correlation, **: negative correlation, *p* < 0.005, Spearman Rho, chi-squared; ^a^ R = 0.058, ^b^ R = 0.041, ^c^ R = −0.041, ^d^ R = 0.081, ^e^ R = −0.042, ^f^ R = −0.039, ^g^ R = 0.042, ^h^ R = −0.069, ^i^ R = 0.042, ^j^ R = −0.043, ^k^ R = −0.044, ^l^ R = 0.038, ^m^ R = 0.044.

**Table 6:** Relationship between genetic mutations and disease severity, renal involvement and AA.

	PRAS Mild	PRAS Modarate	PRAS Severe	Attack Number > 4/Year at the Time of Diagnosis	Renal Involvement wo AA	AA	Colchicine Resistance
M694V het/hom group	116 (16.1%)	249 (34.5%)	356 (49.4%) *^,a^	517 (71.7%)	63 (8.7%) *^,h^	15 (2.1%) *^,i^	60 (8.3%) *^,j^
Compound heterozygote (with M694V)	79 (18.2%)	171 (39.3%)	185 (42.5%) *^,b^	314 (72.2%)	18 (4.1%)	2 (0.5%)	20 (4.6%)
Compound heterozygote (without M694V)	44 (28%)	53 (33.8%)	60 (38.2%)	110 (70.1)	7 (4.5%)	0	8 (5.1%)
E148Q het/hom	63 (31.2%)	81 (40.1%)	58 (28.7%) **^,c^	143 (70.8)	12 (5.9%)	1 (0.5%)	11 (5.4%)
M680I. het/hom	24 (17.3%)	48 (34.5%)	67 (48.2%)	110 (79.1)	11 (7.9%)	2 (1.4%)	9 (6.5%)
V726A het/hom	39 (30.2%)	50 (38.8%)	40 (31%) **^,d^	78 (60.5%) **^,g^	3 (2.3%)	0	6 (4.7%)
R202Q het/hom	19 (22.1%)	30 (34.9%)	37 (43%)	67 (77.9%)	2 (2.3%)	0	1 (1.2%)
Komplex heterozigot	26 (23.4%)	52 (46.8%)	33 (29.7%)	72 (64.9)	5 (4.5%)	2 (1.8%)	2 (1.8%)
Rare mutations	33 (31.7%)	44 (42.3%)	27 (26%)	64 (61.5%)	6 (5.8)	1 (1%)	4 (3.8%)
A744S het/hom	5 (23.8%)	7 (33.3%)	9 (42.9%)	15 (71.4%)	1 (4.8%)	1 (4.8%)	0
R761H het/hom	12 (33.3%)	17 (47.2%)	7 (19.4%) **^,e^	22 (61.1%)	1 (2.8%)	0	3 (8.3%)
P396S het/hom	5 (31.3)	6 (37.5%)	5 (31.3%)	8 (50%)	1 (6.3%)	0	0
K695R het/hom	4 (40%)	5 (50%)	1 (10%) **^,f^	4 (40%)	2 (20%)	0	0
No mutation	105 (33%)	116 (36.5%)	97 (30.5%)	202 (63.5%)	18 (5.7%)	3 (0.9%)	24 (7.5%)

*: positive correlation, **: negative correlation, *p* < 0.005, Spearman Rho, chi-squared; ^a^ *R* = 0.137, ^b^ *R* = 0.042, ^c^ *R =* −0.077, ^d^ *R* = −0.05, ^e^ *R* = −0.051, ^f^ *R =* −0.04, ^g^ *R =* −0.057, ^h^ *R =* 0.074, ^i^ *R =* 0.063, ^j^ *R =* 0.06.

## Data Availability

All data are available upon request.

## References

[1] Arpacı A., Doğan S., Erdoğan H.F., El Ç., Cura S.E. (2021). Presentation of a new mutation in FMF and evaluating the frequency of distribution of the MEFV gene mutation in our region with clinical findings. Mol. Biol. Rep..

[2] Grossman C., Kassel Y., Livneh A., Ben-Zvi I. (2019). Familial Mediterranean fever (FMF) phenotype in patients homozygous to the MEFV M694V mutation. Eur. J. Med. Genet..

[3] Soriano A., Pras E. (2014). Familial mediterranean fever: Genetic update. Isr. Med. Assoc. J..

[4] Gupta N., Kaur H., Wajid S. (2020). Renal amyloidosis: An update on diagnosis and pathogenesis. Protoplasma.

[5] Twig G., Livneh A., Vivante A., Afek A., Shamiss A., Derazne E., Tzur D., Ben-Zvi I., Tirosh A., Barchana M. (2014). Mortality risk factors associated with familial Mediterranean fever among a cohort of 1.25 million adolescents. Ann. Rheum. Dis..

[6] Tanatar A., Sönmez H.E., Karadağ Ş.G., Çakmak F., Çakan M., Demir F., Sozeri B., Aktay N. (2020). Performance of Tel-Hashomer, Livneh, pediatric and new Eurofever/PRINTO classification criteria for familial Mediterranean fever in a referral center. Rheumatol. Int..

[7] Yalçinkaya F., Özen S., Özçakar Z.B., Aktay N., Çakar N., Düzova A., Kasapçopur Ö., Elhan A.H., Doğanay B., Ekim M. (2009). A new set of criteria for the diagnosis of familial Mediterranean fever in childhood. Rheumatology.

[8] Pras E., Livneh A., Balow J.E., Pras E., Kastner D.L., Pras M., Langevitz P. (1998). Clinical Differences Between North African and Iraqi Jews with Familial Mediterranean Fever. Am. J. Med. Genet..

[9] Hentgen V., Grateau G., Kone-Paut I., Livneh A., Padeh S., Rozenbaum M., Amselem S., Gershoni-Baruch R., Touitou I., Ben-Chetrit E. (2013). Evidence-based recommendations for the practical management of Familial Mediterranean Fever. Semin. Arthritis Rheum..

[10] Siligato R., Gembillo G., Calabrese V., Conti G., Santoro D. (2021). Amyloidosis and glomerular diseases in familial mediterranean fever. Medicina.

[11] Touitou I. (2001). The spectrum of Familial Mediterranean Fever (FMF) mutations. Eur. J. Hum. Gen..

[12] Beheshtian M., Izadi N., Kriegshauser G., Kahrizi K., Mehr E.P., Rostami M., Hosseini M., Azad M., Montajabiniat M., Kariminejad A. (2016). Prevalence of common MEFV mutations and carrier frequencies in a large cohort of Iranian populations. J. Genet..

[13] Hageman I.M.G., Visser H., Veenstra J., Baas F., Siegert C.E.H. (2019). Familial Mediterranean Fever (FMF): A single centre retrospective study in Amsterdam. Neth. J. Med..

[14] Dundar M., Emirogullari E.F., Kiraz A., Taheri S., Baskol M. (2011). Common Familial Mediterranean Fever gene mutations in a Turkish cohort. Mol. Biol. Rep..

[15] Yilmaz E., Ozen S., Balci B., Duzova A., Topaloglu R., Besbas N., Saatci U., Bakkaloglu A., Ozguc M. (2001). Mutation frequency of Familial Mediterranean Fever and evidence for a high carrier rate in the Turkish population. Eur. J. Hum. Genet..

[16] Tunca M., Ozdogan H., Kasapcopur O., Yalcinkaya F., Ozen S., Tutar E., Topaloglu R., Yilmaz E., Arici M., Bakkalogu A. (2005). Familial Mediterranean Fever (FMF) in Turkey: Results of a nationwide multicenter study. Medicine.

[17] Kandur Y., Kocakap D.B.S., Alpcan A., Tursun S. (2022). Clinical significance of MEFV gene variation R202Q. Clin. Rheumatol..

[18] Milenković J., Vojinović J., Debeljak M., Toplak N., Lazarević D., Avčin T., Jevtović-Stoimenov T., Pavlović D., Bojanić V., Milojković M. (2016). Distribution of MEFV gene mutations and R202Q polymorphism in the Serbian population and their influence on oxidative stress and clinical manifestations of inflammation. Pediatr. Rheumatol..

[19] Yigit S., Karakus N., Tasliyurt T., Kaya S.U., Bozkurt N., Kisacik B. (2012). Significance of MEFV gene R202Q polymorphism in Turkish familial Mediterranean fever patients. Gene.

[20] Dogan H., Bayrak O.F., Emet M., Keles M., Gulluoglu S., Gul Z., Pirim I. (2015). Familial Mediterranean fever gene mutations in north-eastern part of Anatolia with special respect to rare mutations. Gene.

[21] Sönmezgöz E., Özer S., Gül A., Yılmaz R., Kasap T., Takcı Ş., Gümüşer R., Demir O. (2019). Clinical and Demographic Evaluation According to MEFV Genes in Patients with Familial Mediterranean Fever. Biochem. Genet..

[22] Soylemezoglu O., Kandur Y., Duzova A., Ozkaya O., Kasapcopur O., Baskin E., Fidan K., Yalcinkaya F. (2015). Familial Mediterranean fever with a single MEFV mutation: Comparison of rare and common mutations in a Turkish paediatric cohort. Clin. Exp. Rheumatol..

[23] Gumus E. (2018). The frequency of MEFV gene mutations and genotypes in Sanliurfa Province, south-eastern region of Turkey, after the syrian civil war by using next generation sequencing and report of a novel exon 4 mutation (I423T). J. Clin. Med..

[24] Mansour A.R., El-Shayeb A., El Habachi N., Khodair M.A., Elwazzan D., Abdeen N., Said M., Ebaid R., ElShahawy N., Seif A. (2019). Molecular patterns of MEFV gene mutations in Egyptian patients with familial mediterranean fever: A retrospective cohort study. Int. J. Inflam..

[25] Fujikura K. (2015). Global epidemiology of familial mediterranean fever mutations using population exome sequences. Mol. Genet. Genom. Med..

[26] Aydın F., Çakar N., Özçakar Z.B., Uncu N., Başaran Ö., Özdel S., Çelikel E., Elhan A.H., Yalçınkaya F. (2019). Clinical features and disease severity of Turkish FMF children carrying E148Q mutation. J. Clin. Lab. Anal..

[27] Kasifoglu T., Bilge S.Y., Sari I., Solmaz D., Senel S., Emmungil H., Kilic L., Oner S.Y., Yildiz F., Yilmaz S. (2014). Amyloidosis and its related factors in Turkish patients with familial mediterranean fever: A multicentre study. Rheumatology.

[28] Akpolat T., Özkaya O., Özen S. (2012). Homozygous M694V as a risk factor for amyloidosis in Turkish FMF patients. Gene.

[29] Ozturk C., Halicioglu O., Coker I., Gulez N., Sutçuoglu S., Karaca N., Aksu G., Kutukculer N. (2012). Association of clinical and genetical features in FMF with focus on MEFV strip assay sensitivity in 452 children from western Anatolia, Turkey. Clin. Rheumatol..

[30] Ben-Chetrit E., Yazici H. (2019). Familial Mediterranean fever: Different faces around the world. Clin. Exp. Rheumatol..

[31] Aktaş A., Karadavut M., Cansu D.Ü., Korkmaz C. (2019). The influence of genotype on disease severity and concomitant diseases in familial Mediterranean fever patients. Clin. Exp. Rheumatol..

[32] Shinar Y., Giat E., Cohen R., Livneh A. (2015). Assessment of the pathogenicity of the p.K695R and p.A744S Mediterranean fever gene variants. Pediatr. Rheumatol..

[33] Ayaz N.A., Tanatar A., Karadağ Ş.G., Çakan M., Keskindemirci G., Sonmez H.E. (2021). Comorbidities and phenotype–genotype correlation in children with familial Mediterranean fever. Rheumatol. Int..

[34] Touitou I., Sarkisian T., Medlej-Hashim M., Tunca M., Livneh A., Cattan D., Yalcinkaya F., Ozen S., Majeed H., Ozdogan H. (2007). Country as the primary risk factor for renal amyloidosis in familial Mediterranean fever. Arthritis Rheum..

[35] Ait-Idir D., Khilan A., Djerdjouri B., El-Shanti H. (2011). Spectrum of mutations and carrier frequency of familial Mediterranean fever gene in the Algerian population. Rheumatology.

[36] Özçakar Z.B., Elhan A.H., Yalçinkaya F. (2014). Can colchicine response be predicted in familial Mediterranean fever patients?. Rheumatology.

[37] Barut K., Sahin S., Adrovic A., Sinoplu A.B., Yucel G., Pamuk G., Aydin A.K., Dasdemir S., Turanli E.T., Buyru N. (2018). Familial Mediterranean fever in childhood: A single-center experience. Rheumatol. Int..

[38] Çakan M., Karadağ Ş.G., Ayaz N.A. (2020). Canakinumab in colchicine resistant familial mediterranean fever and other pediatric rheumatic diseases. Turk. J. Pediatr..

[39] Yücel B.B., Aydog O., Nalcacioglu H., Yılmaz A. (2021). Effectiveness of Canakinumab Treatment in Colchicine Resistant Familial Mediterranean Fever Cases. Front. Pediatr..

